# Short-Term High Fructose Intake Impairs Diurnal Oscillations in the Murine Cornea

**DOI:** 10.1167/iovs.62.10.22

**Published:** 2021-08-20

**Authors:** Jingxin He, Xinwei Jiao, Xin Sun, Yijia Huang, Pengyang Xu, Yunxia Xue, Ting Fu, Jun Liu, Zhijie Li

**Affiliations:** 1 International Ocular Surface Research Center, Institute of Ophthalmology and Key Laboratory for Regenerative Medicine, Jinan University, Guangzhou, China; 2 Department of Ophthalmology, The First Affiliated Hospital of Jinan University, Guangzhou, China; 3 Department of Pathophysiology, Jinan University Medical School, Guangzhou, China

**Keywords:** bioinformatics, circadian clock, cornea, fructose, RNA sequencing

## Abstract

**Purpose:**

Endogenous and exogenous stressors, including nutritional challenges, may alter circadian rhythms in the cornea. This study aimed to determine the effects of high fructose intake (HFI) on circadian homeostasis in murine cornea.

**Methods:**

Corneas of male C57BL/6J mice subjected to 10 days of HFI (15% fructose in drinking water) were collected at 3-hour intervals over a 24-hour circadian cycle. Total extracted RNA was subjected to high-throughput RNA sequencing. Rhythmic transcriptional data were analyzed to determine the phase, rhythmicity, unique signature, metabolic pathways, and cell signaling pathways of transcripts with temporally coordinated expression. Corneas of HFI mice were collected for whole-mounted techniques after immunofluorescent staining to quantify mitotic cell number in the epithelium and trafficking of neutrophils and γδ-T cells to the limbal region over a circadian cycle.

**Results:**

HFI significantly reprogrammed the circadian transcriptomic profiles of the normal cornea and reorganized unique temporal and clustering enrichment pathways, but did not affect core-clock machinery. HFI altered the distribution pattern and number of corneal epithelial mitotic cells and enhanced recruitment of neutrophils and γδ-T cell immune cells to the limbus across a circadian cycle. Cell cycle, immune function, metabolic processes, and neuronal-related transcription and associated pathways were altered in the corneas of HFI mice.

**Conclusions:**

HFI significantly reprograms diurnal oscillations in the cornea based on temporal and spatial distributions of epithelial mitosis, immune cell trafficking, and cell signaling pathways. Our findings reveal novel molecular targets for treating pathologic alterations in the cornea after HFI.

##  

Global sugar consumption has increased substantially over the last 30 years and constitutes 15% to 17% of total daily calorie consumption in Western diets.^1^^–^^3^ Sugars are present in most processed foods and as sweeteners in soft drinks. Extant data suggest that the overconsumption of sugar is associated with a rapid increase in the onset, prevalence, and development of the metabolic syndrome, obesity, diabetes, cardiovascular disease, and cancer.^4^^–^^6^ This global public health issue poses a considerable social and economic burden in both developing and industrialized countries.^7^^,^^8^ Thus, exploring the mechanisms underlying the effects of excessive sugar consumption on human health is critical for the optimization of dietary practices and nutritional intervention strategies.^9^

The impact of high sugar consumption on eye health is a topic of growing interest.^10^^–^^12^ Data obtained from the large-scale Age-Related Eye Disease Study revealed that decreasing the amount of added sugars and refined carbohydrates to the diet attenuated the risk and progression of AMD in populations at high risk of this blinding eye condition.^13^^,^^14^ An increase in carbohydrate consumption has been associated with the occurrence of cataracts.^15^ A high sugar concentration in the lens promotes cataract formation via protein damage and clumping.^16^^,^^17^ Further, high-sugar diets have been linked to the occurrence of dry eyes. Clinical observations suggest that the consumption of high-sugar diets aggravates dry eye symptoms.^18^

Dietary carbohydrates, especially fructose, are major contributors to the development of systemic complications.^19^ In contrast with glucose metabolism, which is tightly regulated and involves hormonal control by insulin, fructose metabolism is less tightly regulated.^19^ At high doses, fructose predominantly results in lipid production and increases the fat burden on the liver.^20^ Indeed, decreasing dietary fructose and sugar consumption in children and adults attenuates liver fat accumulation and improves cardiovascular and diabetes risk markers.^21^ Collectively, these data highlight the metabolic stress induced by high fructose intake (HFI). The cornea is an active tissue that undergoes renewal and metabolism.^22^^,^^23^ Nevertheless, the effects of HFI on metabolism in the cornea remain unclear.

Metabolic processes and circadian rhythms are closely interlinked.^24^ In mammals, almost all physiological functions exhibit circadian rhythms according to a 24-hour day based on the earth's light/dark cycle.^25^ This adaptation is underscored by orchestrated rhythms in cellular and molecular processes in the suprachiasmatic nucleus (SCN) of the hypothalamus as a central pacemaker. The SCN is entrained by the light/dark cycle via the retino-hypothalamic tract and synchronizes with peripheral clocks in peripheral organs or tissues. In addition to light as a zeitgeber (daytime cue), other SCN-independent zeitgebers, including dietary nutrients,^26^ feeding time,^27^ and intake of drugs of abuse^28^ can alter circadian rhythms. Converging evidence suggests that under normal light/dark cycles, the consumption of a high-calorie diet affects the circadian rhythms of critical organs such as the liver and brain at the transcriptional and proteomic levels.^29^^–^^31^

The cornea is located at the front of the eyeball. The maintenance of the normal transparent state of the cornea is essential for the accurate projection of external light onto the retina and the formation of clear images. However, corneal homeostasis is influenced by various exogenous and endogenous factors. Indeed, corneal growth and development are affected by light/dark cycles.^32^ Exposure to constant light, constant dark, and jet lag alter the mitotic cell number and expression of core-clock genes in the corneal epithelium.^33^ Corneal thickness and transcriptome also exhibit diurnal fluctuations.^34^^,^^35^ Streptozotocin-induced hyperglycemia significantly attenuates the mitotic division rhythms in the corneal epithelium and promotes circadian recruitment of peripheral neutrophils from the blood circulation to the limbus in mice.^36^ These findings indicate that both endogenous and exogenous stress, including nutritional challenges, may alter circadian rhythms in the cornea.

Given the excessive fructose intake in modern diets, this study aimed to examine the effects of short-term HFI on circadian rhythms in the cornea. We hypothesized that the consumption of a high fructose diet would affect circadian rhythms in the cornea at the transcriptomic level. To test this hypothesis, we employed high-throughput RNA sequencing (RNA-seq) and evaluated corneal epithelial mitosis and immune cell recruitment to examine the temporal and spatial effects of HFI on circadian oscillations in the cornea.

## Methods

### Study Design

**Figure 1.**
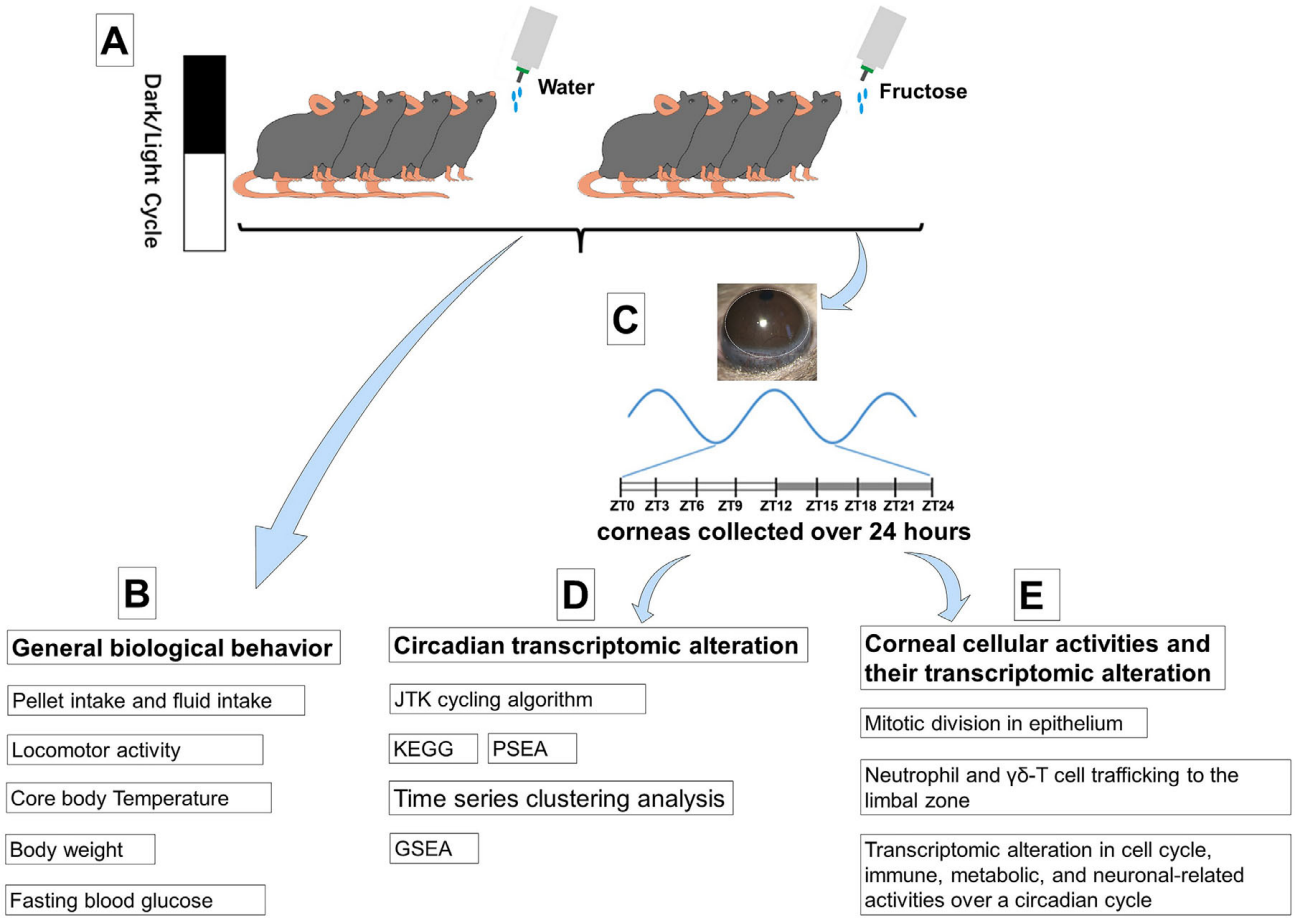
Experimental setup and data analysis flowchart. (**A**) After 2 weeks of acclimatization to a 12-hour light/12-hour dark cycle, mice were randomly divided into NC (sterile water only) or HFI groups (10-day exposure to HFI). (**B**) After the HFI protocol, consummatory behaviors and physiological parameters of NC and HFI mice were recorded and compared at different time points. (**C**) On day 10 after the HFI protocol, the corneas of NC and HFI mice were collected at 3-hour intervals at eight different time points over a 24-hour cycle, indicated in ZT. (**D**) On day 10, after the HFI protocol, extracted RNA from corneas of NC and HFI mice was processed for RNA-seq data. JTK algorithm, KEGG analysis, PSEA, and time series clustering analysis were used to determine the circadian transcriptional landscape of daily rhythmic genes. GSEA was used to determine whether an a priori defined set of genes exhibited a statistically significant difference between corneas of NC and HFI mice. (**E**) On day 10 after the HFI protocol, the number of mitotic cells in the corneal epithelium and number of recruited immune cells (neutrophils and γδ-T cells) to the corneal limbus over a 24-hour cycle were quantified.

The experimental design and analysis are depicted in Figure 1. General behavior profiling highlighted essential physiological features, including food and fluid intake, body weight, locomotor activity, core body temperature, and plasma glucose concentration (Fig. 1B). Circadian transcriptomic profiling involved RNA-seq followed by an investigation of circadian transcriptomic analysis (including phase, amplitude, and periodicity), rhythmic transcript identification using the JTK cycling algorithm, phase set enrichment analysis (PSEA), gene set enrichment analysis (GSEA), and time series clustering analysis (Figs. 1C and D). Cellular activity-associated transcriptomic profiling was performed to evaluate corneal cellular activity. Mitotic division in the corneal epithelium and recruitment of neutrophils and γδ-T cells to the corneal limbus over a circadian cycle were assessed (Fig. 1E).

### Animals

All animal care and experimental use followed the guidelines described in the ARVO Statement for the Use of Animals in Vision and Ophthalmic Research and was approved by the Jinan University Institutional Animal Care and Use Committee (JN-A-2002-01). Male C57BL/6J mice at 8 to 10 weeks of age were obtained from the Medical Experimental Animal Center (Guangdong, China) and were housed in light-tight circadian chambers (Longer-Biotech Co., Ltd, Guangzhou, China). Time was indicated using the zeitgeber time (ZT) scale as an indicator of rhythm phase, whereby ZT0 and ZT12 referred to the time of lights on (7 am) and lights off (7 pm), respectively. Mice were provided ad libitum access to a standard chow diet and water throughout the experimental period. Animals were euthanized by isoflurane overdose inhalation and cervical dislocation.

### HFI Protocol

The HFI protocol was conducted as previously described.^11^ Briefly, mice were divided randomly into two groups (Fig. 1A). The HFI group comprised 8-week-old animals that were provided a standard pellet diet and sterile tap water containing 15% D(-)-fructose (g/0.1 L) for 10 days. The normal control (NC) group comprised age-matched mice that were provided a standard pellet diet and sterile tap water. Pellet intake, fluid intake, and body weight were measured every 2 days at ZT6. Body weight was measured at the beginning and end of the experiments. Blood glucose concentrations were assayed from the tail tip of each mouse. Fasting blood samples were collected and measured using a glucometer (Accu-Chek Active glucometer, Roche, Germany). After 10 days of exposure to HFI, mice were fasted from ZT4, and tail blood samples were collected at ZT10. Hyperglycemia was defined as blood glucose concentrations of 11.10 mmol/L or more.

### Behavioral Analysis

The locomotor activity of individually housed mice was measured using the Mini Mitter telemetry system (Mini Mitter, Bend, OR) as previously described.^37^ Animals were implanted with a PDT-4000 E-Mitter (Mini Mitter) into the peritoneal cavity under pentobarbital sodium anesthesia (80 mg/kg of body weight, intraperitoneally) (Sigma-Aldrich, St Louis, MO). Data were collected over 20-minute and 5-minute intervals for core body temperature and locomotion, respectively.

Corneal sensitivity was determined as previously described.^38^ Briefly, a monofilament with a Bonnet esthesiometer (catalog no. 8630-1490-29; Luneau Technology, Pont-del-l'Arche, France) was used to perpendicularly contact the central corneal area four times. The monofilament length was recorded as the sensitivity index using a double-blind approach based on the blink reflex.

### Wholemount Technique and Immunohistochemistry of Murine Cornea

After euthanasia, both corneas were removed as previously described.^33^^,^^39^^–^^42^ In brief, corneas with complete limbi were fixed in 2% paraformaldehyde in PBS for 40 minutes, subjected to three 5-minute washes in PBS, blocked in 0.1 M PBS containing 2% BSA for 15 minutes, and permeabilized with 0.1% Triton X-100 in BSA/PBS for 15 minutes. Subsequently, corneas were incubated in 0.1 M BSA/PBS and 0.1% Triton X-100 with anti-Ly6g FITC (3:100; BD Biosciences, San Jose, CA) to detect neutrophils, anti–CD31-PE (3:100, BD Biosciences) to detect limbal vessels, or anti–TCRδ-PE (clone GL3, 3:100, BD PharMingen, Franklin Lake, NJ) for 24 hours at 4 °C. After incubation, tissues were subjected to three 5-minute washes in 0.1 M PBS. Whole corneas were sectioned into four quadrants, stretched, and mounted on slides using anti-fade mounting media with 1 µM 4,6-diamidino-2-phenylindole (DAPI) (Sigma-Aldrich) overnight, and stored in the dark until further analysis.

### Quantification of Mitotic Corneal Epithelial Cells and Immune Cells

**Figure 2.**
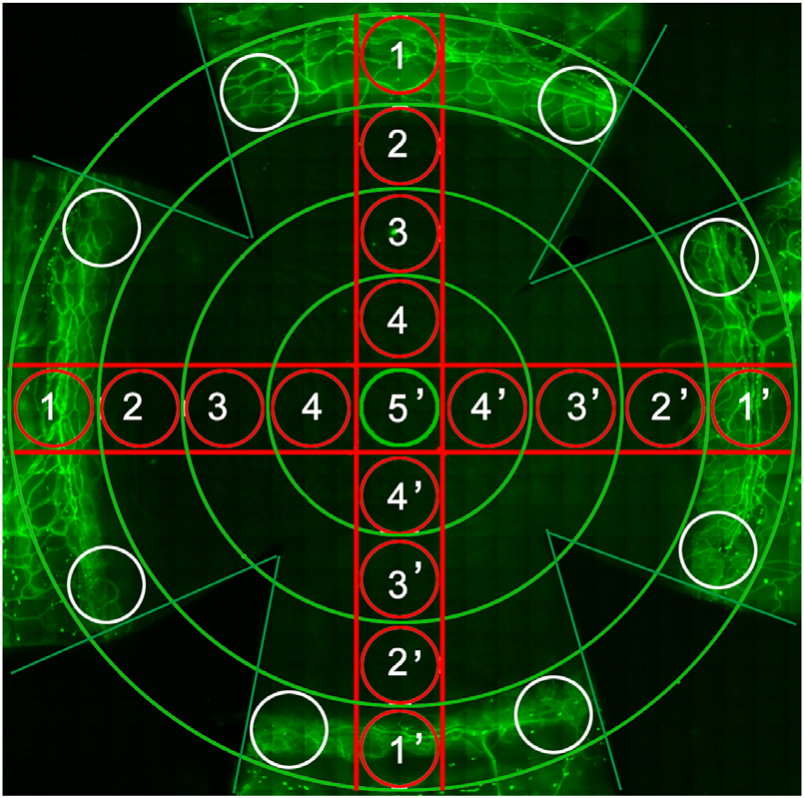
Diagram depicting microscopic fields for quantitative cellular analysis. The background is a representative whole-mount corneal image collected and stitched using a 40× DeltaVision Elite microscope. The red circles across the cornea in nine 40× fields comprised fields 1 to 5 and 5′ to 1′ for mitotic cell quantification in the epithelium. The eight white circles in the limbus were used to quantify neutrophils around limbal vessels (*in green*) and γδ-T cells around limbal vessels and in the epithelium from fields 1 through 3 in the *red circles*.

Corneas with complete limbi were collected at 3-hour intervals over a circadian cycle and were mounted on glass slides as previously described.^36^^,^^43^^,^^44^ To quantify mitotic cell number in the corneal epithelium, two corneal diameters were selected, and the number of DAPI-stained and paired nuclei were quantified between each side of the limbus using a DeltaVision Image System with a 40× magnification field (Fig. 2, red circle-containing lines). Neutrophils surrounding the limbal vessels were quantified in eight different regions of the corneal limbus at 40× magnification (Fig. 2, white circles). The γδ-T cells were quantified from the limbus to central corneal direction (from fields 1 to 3 in red circles) in the corneal epithelium and stroma.

### Oil Red O Staining

Eyeballs and livers were frozen over liquid nitrogen, embedded in optimal cutting temperature compound (Tissue-Tek Compound 4583, Sakura, Torrance, CA), and stained with routine Oil Red O (Cat # G1016, Servicebio Company, Wuhan, China) as previously described.^45^^,^^46^ Images were acquired at 20× magnification using a slide scanner (Pannoramic 250/MIDI, 3D HISTECH Ltd, Budapest, Hungary).

### Tissue Sample Collection and RNA Extraction

On day 10, after the conclusion of the HFI regimen, corneas were collected at 3-hour intervals over a circadian cycle as described previously.^35^ Samples were rapidly frozen over liquid nitrogen. Total RNA was isolated from two pooled corneas from each animal using a Trizol RNA extraction protocol followed by cleanup using the RNeasy spin column kit (Qiagen, Hilden, Germany). All sample collections were completed within a 2-week period in January 2017 to avoid the effects of seasonal changes.

### RNA-Seq

RNA-seq was performed as previously described.^11^^,^^35^ Briefly, RNA purity was determined using NanoDrop (Waltham, MA, USA). RNA concentration was measured using Qubit 2.0 Fluorometer (Life Technologies, Carlsbad, CA). RNA integrity was verified using an Agilent 2100 instrument (Santa Clara, CA). Construction and sequencing of the cDNA library were performed by the Beijing Genomics Institute (BGI) using the BGISEQ-500 platform according to the manufacturer's protocol. Each sample produced more than 20 M clean reads, which were mapped to the mm10 reference genome version using Spliced Transcripts Alignment to a Reference (STAR 2.5.3a).

### Analysis of Rhythmic Gene Expression

To map the global effects of short-term HFI on circadian transcript expression in the cornea, we collected corneas from NC and HFI mice every 3 hours over a 24-hour circadian cycle, extracted RNA from three biological replicates, and performed individual RNA-seq to high depth (Fig. 1C and E). Analysis of rhythmic gene expression in the cornea was performed as previously described.^11^^,^^35^ Briefly, time-ordered fragments per kilobase of transcript per million mapped reads (FPKM) of actively transcribed genes were triplicated and input into the JTK_CYCLE algorithm in the R package to detect rhythmic components in genome-scale datasets over eight time points.^47^ Between-sample normalization was performed using the DESeq median normalization method. FPKM mapped reads were calculated to obtain measurements of relative gene expression within and between biological samples. Oscillating transcripts were defined as those with a JTK_CYCLE *P* value of less than 0.05 and an oscillation period within a 24-hour range.^48^ Based on the adjusted *P* value from the JTK_CYCLE output, we identified circadian transcription patterns in the corneas of NC and HFI mice. Circadian expression patterns were evaluated according to the direction (µ) and length (r) of the mean vector, which represented the average of all phases and degree of synchronization, respectively.

### Functional Annotation With Kyoto Encyclopedia of Genes and Genomes (KEGG)

Enriched pathways from KEGG for circadian genes against the background of expressed transcripts in the corneas of NC and HFI mice were annotated as previously described.^11^^,^^35^ The resulting annotations were grouped into annotation clusters based on common gene members. The enrichment score (ES) of each annotation cluster was defined as the geometric mean (in a –log10 scale) of the nonadjusted *P* values (*P* < 0.05) of individual annotations. To examine the relationships between specific rhythmic genes and KEGG pathways, KEGG network diagrams were visualized using a BGI in-house customized data mining system termed Dr. Tom (http://biosys.bgi.com).

### GSEA

To characterize signaling pathways associated with HFI, GSEA was performed through the GSEA software (v4.1.0, http://www.broadinstitute.org/gsea/index.jsp, Broad Institute at MIT, Cambridge, MA) using format-converted mouse RNA-seq data as the expression dataset as previously described.^49^ The database of analyzed gene sets (c2.cp.v7.4.symbols.gmt) included validated hallmark signatures derived from the Molecular Signatures Database and reflected transcriptional programs involved in metabolism, infection, immunity, and the cell cycle. We evaluated enrichment in phenotypes exhibiting positive or negative correlations with HFI. Gene expression in each model system was ranked according to real fold-change expression relative to corresponding controls. GSEA was performed using default parameter settings. The visualization of GSEA results is mainly divided into the following three parts^50^: (1) the ES, which reflects the degree to which a gene set is overrepresented at the extremes (top or bottom) of the entire gene ranked list. Higher the ES value is, more enriched the pathway in the sample; (2) a ranked gene list—each black vertical line below the ES fold line represents a gene in the functional gene set and its location in the sorted gene list after phenotypic association ranking; and (3) a heatmap—the left red part of the heatmap at the bottom represents the high expression of the corresponding functional pathway genes in the HFI group, and the right blue part represents the high expression of the corresponding functional pathway genes in the NC group. A positive normalized ES indicates higher expression in the HFI than in the NC group. A nominal *P* value was used to characterize the credibility of the enrichment results. It is generally considered that the pathways with a normalized ES of more than 1 and a *P* value of less than 0.05 are significantly enriched.

### PSEA

Circadian pathways were characterized with PSEA (Software version 1.1), downloaded from https://omictools.com/psea-2-tool, based on circadian transcript sets.^51^ Gene sets of “c2.cp.kegg.v6.2.symbols.gmt” were downloaded from the Molecular Signatures Database C2 (KEGG gene sets).^49^ The parameters were set to domains from 0 to 24 (phases between 0 and 24) and enrichment of each pathway in more than 10 genes, with a Kuiper *Q* value of less than 0.01 and max sims per test of 10,000.

### Time Series Clustering Analysis

To visualize the trends in transcriptional expression in the cornea over time, noise-robust soft clustering analysis was performed using the fuzzy c-means clustering algorithm in the Mfuzz package (http://www.bioconductor.org/packages/release/bioc/html/Mfuzz.html).^52^^,^^53^ As in our previously described protocol,^54^^,^^55^ all rhythmic genes from NC and HFI-treated corneas with eight timepoint gradients using the JTK_CYCLE were first imported into the algorithm, respectively. The R package default was set with 0.7 as the core threshold (i.e., membership > 0.7 was considered a cluster core). Second, four specific clusters were selected based on cycling gene expression trends in the corneas of NC and HFI animals. Third, to understand the dynamic pattern of these genes and their relationship with function, we also performed KEGG pathway enrichment analysis on these rhythmic genes contained in each cluster.

### Statistical Analysis and Software

GraphPad Software (GraphPad Prism 8.0; La Jolla, CA) was used for the generation of bar, scatter, and line charts; violin plotting; and statistical analysis. Oriana software (Version 4.01; Kovach Computing Services, Pentraeth, Wales, UK) was used to analyze the phase, period distribution, and Rayleigh vector of oscillating genes. The Venn Diagram Plotter (Venny 2.1.0, http://bioinfogp.cnb.csic.es/tools/venny/index.html) was used to compare the numbers of rhythmic genes in different groups. Heatmaps were generated using pheatmap scripts in R (64-bit, version 3.6.1). The normality of all data was determined by the Shapiro–Wilk test to examine the homogeneity of variance. Between-group comparisons were performed using the Student *t*-test or one-way ANOVA with Bonferroni correction for multiple comparisons. Data are presented as mean ± standard error of the mean for quantitative variables and as frequencies for classification variables. *P* values of less than 0.05 were considered statistically significant; n.s. indicates not statistically significant.

## Results

### HFI Alters Consummatory Behaviors and Physiological Parameters

**Figure 3.**
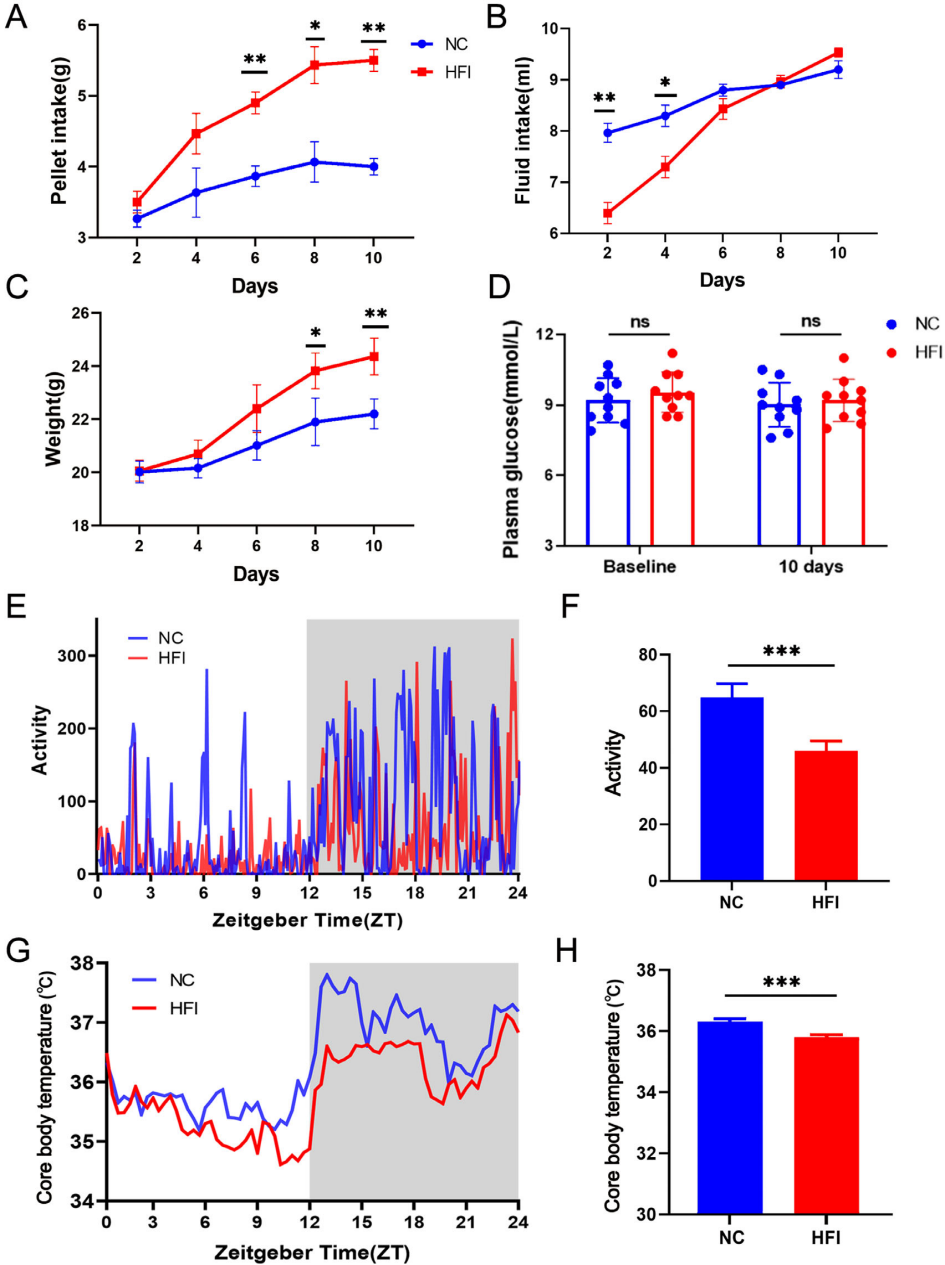
Behavioral observations of NC and HFI mice. (**A**) Pellet intake in NC mice (*blue*) and HFI mice (*red*) during the 10 days after the start of HFI. **P < 0.01; ***P < 0.001, *n* = 10 mice per group. (**B**) Fluid intake in NC mice (*blue*) and HFI mice (*red*) during the 10 days after the start of HFI. *P < 0.05; **P < 0.01; n = 10 mice per group. (**C**) Kinetics of body weight gain in NC mice (*blue*) and HFI mice (*red*) during the 10 days after the start of HFI. *P < 0.05; **P < 0.01; n = 10 mice per group. (**D**) Plasma glucose levels in NC mice (*blue*) and HFI mice (*red*) on day 10 after the start of HFI. n.s., *n* = 10 mice per group. (E) Representative locomotor activity over a circadian cycle in a single mouse on day 10. The *blue and red lines* indicate NC and HFI mice, respectively. The *gray shading* indicates dark cycles. (**F**) Mean overall activity of NC and HFI mice over a circadian cycle. (**G**) Representative core body temperature rhythms over a circadian cycle in a single mouse on day 10. The *blue and red lines* indicate changes in NC and HFI mice, respectively. The *gray shading* indicates circadian dark phases. (**H**) The overall mean core body temperature of NC and HFI mice over a circadian cycle.

A significant difference was observed in pellet intake after day 6 after HFI between the NC and HFI mice (Fig. 3A). The fluid intake of HFI-treated mice was significantly decreased on day 2 and day 4 after the initiation of feeding compared with that of NC-fed animals (Fig. 3B). Body weight was significantly higher in HFI mice than in NC animals on days 8 and 10 after HFI (Fig. 3C). No significant between-group differences were observed in plasma glucose levels after a 6-hour fast (Fig. 3D), consistent with our previous findings.^11^ To determine the effects of short-term HFI on circadian behavior, we implanted mice with telemeters and tracked core body temperature and physical activity as previously described.^54^ Core body temperature and locomotor activity in HFI animals exhibited normal daily oscillations, but the amplitudes of both oscillations were lower than those in NC animals (Figs. 3E–H).

### HFI Alters Corneal Transcriptome Composition

**Figure 4.**
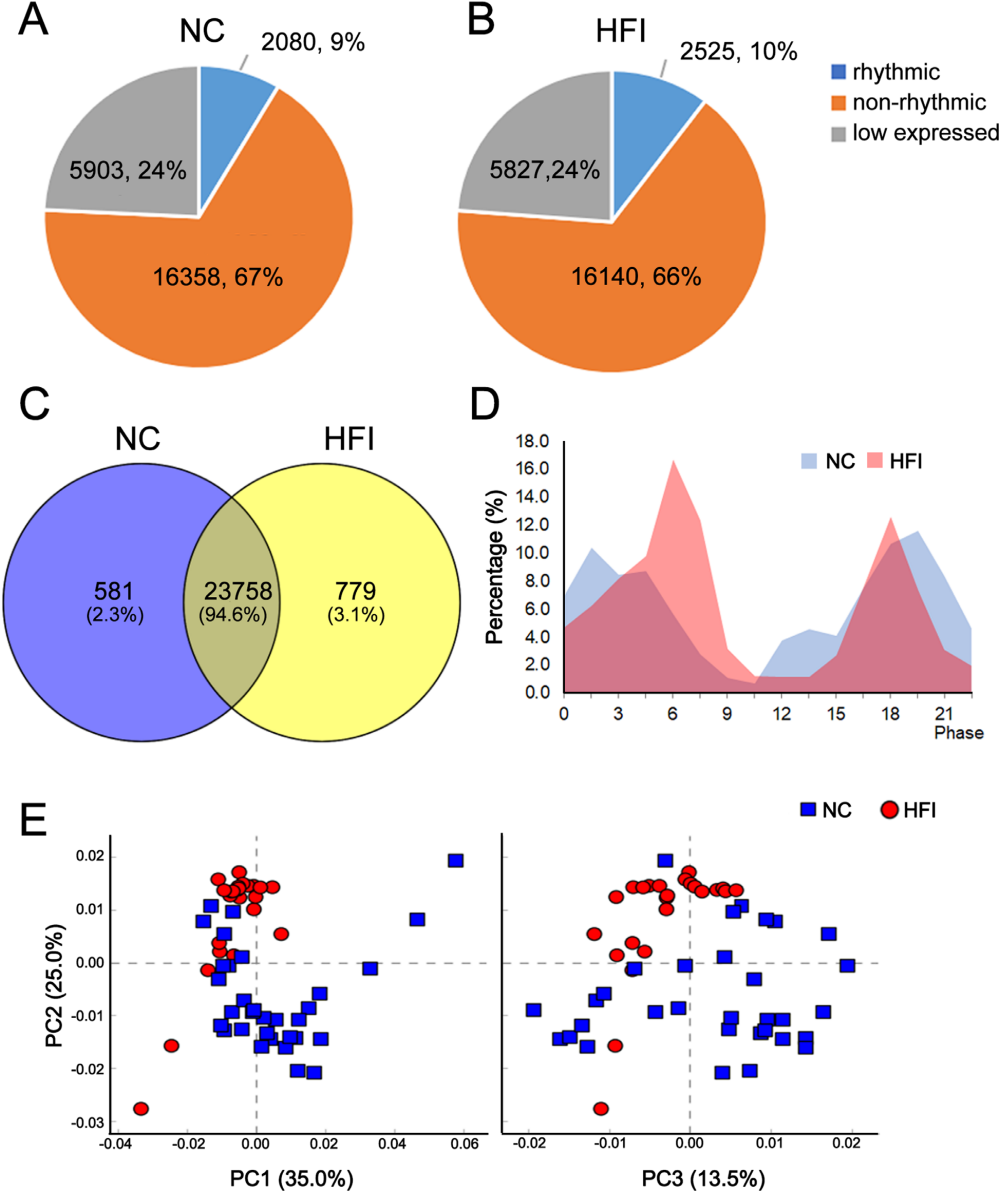
Alterations in transcriptome composition in the corneas of NC and HFI mice. (**A** and **B**) Pie charts of transcriptome composition in the corneas of NC and HFI mice. (**C**) Venn diagram representing the overlapping sets of transcripts (FPKM > 0) in the corneas of NC and HFI mice. (**D**) Distribution of peak gene expression in the corneas of NC and HFI mice over different ZT time points in a circadian cycle. (**E**) Principal component analysis scatterplot of PC1, PC2, and PC3. *Blue and red dots* represent the NC and HFI groups, respectively.

To identify different transcriptome patterns in the corneas of NC and HFI mice, we divided the entire transcriptome into three categories: (1) genes with expression that varied over a 12-hour light/12-hour dark cycle (rhythmic genes; JTK algorithm, adjusted *Q* value < 0.05; FPKM ≥ 0.1), (2) genes with expression that remained consistent over the 24-hour period (nonrhythmic genes; JTK algorithm, adjusted *Q* value ≥ 0.05; FPKM ≥ 0.1), and (3) low-expression genes (FPKM < 0.1), as previously described.^35^ Among the 24,341 transcripts detected in corneas of NC mice, rhythmic transcripts accounted for 9% (2080), nonrhythmic transcripts for 67% (16,358), and low-expression transcripts for 24% (5903). The 24,492 transcripts detected in corneas of HFI mice exhibited a similar pattern, constituting 10% (2525) rhythmic genes, 66% (16,140) nonrhythmic genes, and 24% (5827) low-expression genes (Fig. 4A and B, Supplementary Table S1). In total, 25,118 nonoverlapping transcripts were detected in the cornea over a 24-hour cycle, of which 23,758 (94.6%) were detected in both groups, 581 (2.3%) solely in NC mice and 779 (3.1%) solely in HFI mice (Fig. 4C).

In the corneas of NC mice, oscillating gene expression exhibited a biphasic profile that peaked at ZT1.5 and ZT19.5. In the corneas of HFI mice, a single sharp peak occurred at ZT6 (Fig. 4D). A comparative analysis of duplicates in RNA-seq data at ZT6 and ZT18 (peak time in the HFI group) was visualized using a volcano plot. A principal component analysis was performed to examine the differences in the corneal transcripts and the variance in each component between groups. In total, 24 variables were analyzed for dimensional reduction. Three principal components were extracted according to the eigenvalue (>1). The variances of the three principal components (PC1, PC2, and PC3) accounted for 35.0%, 25.0%, and 13.5% of the total, respectively. The standardized load coefficients of 24 variables in each principal component for both groups are presented in Figure 4E. In NC mice, 24 variables correlated positively with PC1 and weakly with PC2 and PC3. Most variables in HFI mice correlated negatively with PC1. Collectively, these data suggest that HFI globally altered corneal transcriptome composition under homeostatic conditions.

### HFI Modulates Global Transcriptional Rhythmic Gene Expression Phase and Amplitude in the Cornea

**Figure 5.**
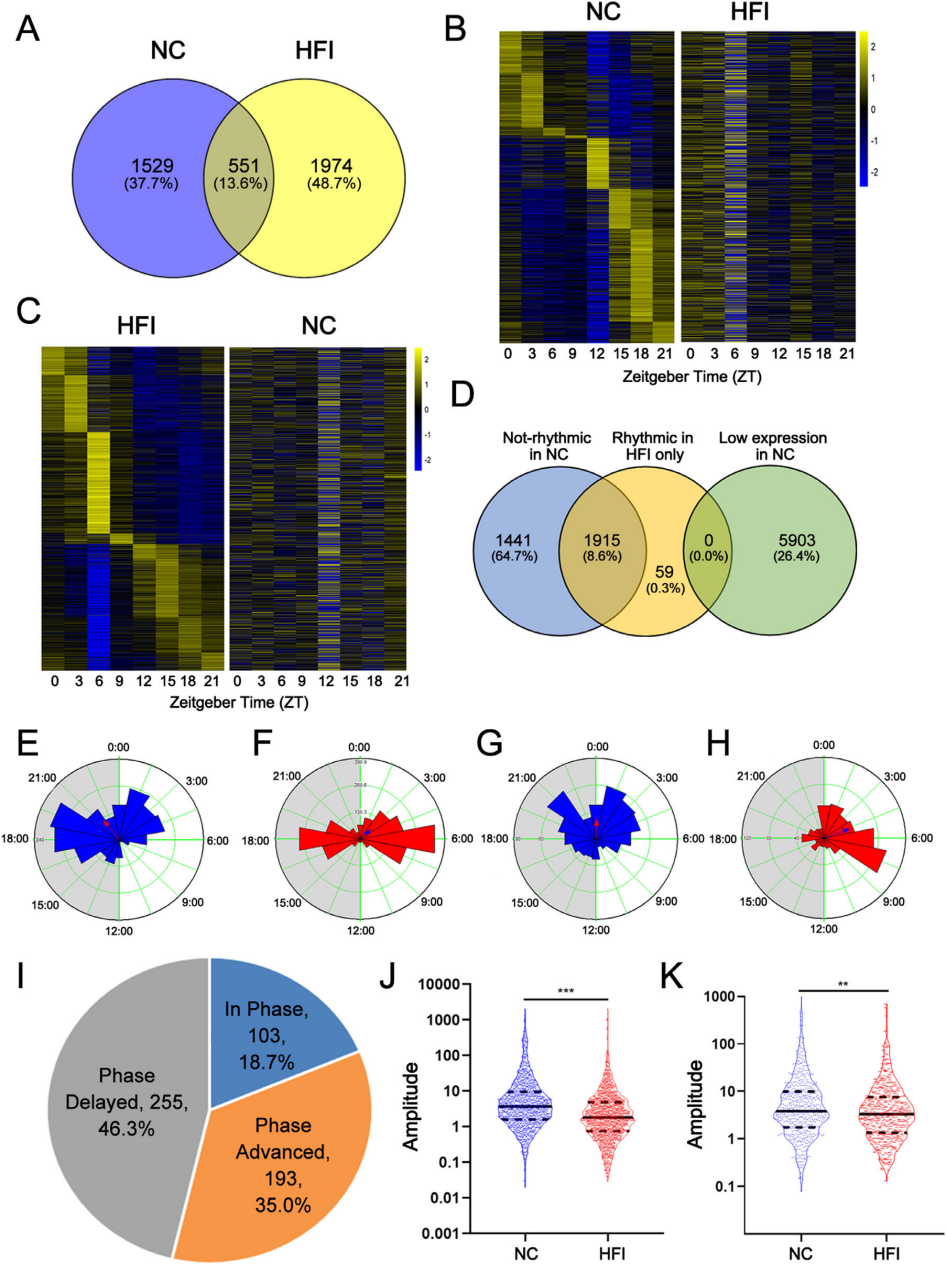
Reprogramming of rhythmic transcriptome in the murine cornea after HFI. (**A**) Venn diagram displaying the total numbers and percentages of rhythmic genes in the corneas of NC and HFI mice. (**B**) Heatmap displaying the 1529 transcripts that were identified to oscillate exclusively in the NC group (*Q* < 0.05). The expression changes over time for these transcripts are presented for the NC group (*left*) and HFI group (*right*). The color bar indicates the scale for transcript expression across eight time points, with the expression range normalized to ±2. (**C**) Heatmap displaying the 1974 transcripts that were identified to oscillate exclusively in the HFI group (*Q* < 0.05). The expression changes over time for these transcripts are presented for the HFI group (*left*) and NC group (*right*). The color bar indicates the scale for transcript expression across eight time points, with the expression range normalized to ±2. (**D**) Venn diagram displaying the overlapping number of rhythmic genes in the corneas of HFI mice with nonrhythmic genes and low-expressed transcripts in corneas of NC mice. (**E–H**) Phase analysis of oscillating genes in the murine cornea. (**E**) NC-specific oscillating genes. (**F**) HFI-specific cycling genes. (**G**) Shared oscillating genes in the NC group. (**H**) shared oscillating genes in the HFI group. The *gray shading* indicates dark cycles. (**I**) Phase analysis of shared genes that retained circadian rhythms in the corneas of NC and HFI mice. (**J**) Oscillation amplitudes of unique oscillating transcripts in the corneas of NC (*left*) and HFI mice (*right*). **P < 0.01. (**K**) Amplitudes of shared cycling gene oscillations in the corneas of NC (*left*) and HFI mice (*right*). **P < 0.01.

Venn and heatmap plotting analyses of these rhythmic genes indicated that 4054 transcripts in the corneas of NC and HFI mice exhibited rhythmic expression. Of these, 37.7% (1529 transcripts) exhibited rhythmic expression only in NC mice (Fig. 5A *left*), 48.7% (1974 transcripts) exhibited rhythmic expression only in HFI mice (Fig. 5A *right*), and 13.6% (551) exhibited rhythmic expression in both groups (Fig. 5A *middle* and Supplementary Table S2). Heatmaps verified the differences in circadian expression among transcript sets identified to be exclusively rhythmic in the corneas of NC (Fig. 5B) and HFI mice (Fig. 5C).

We further examined whether the 1974 transcripts that were exclusively rhythmic in HFI mice fell into nonrhythmic or low-expression categories in NC mice. Venn plotting revealed that all of the transcripts that were exclusively rhythmic in HFI mice fell into the nonrhythmic category in NC mice (Fig. 5D), indicating that HFI-induced rhythmic transcripts were nonrhythmically expressed in the corneas of NC mice. We plotted the phase, period, and Rayleigh vector of oscillating genes for shared, NC-specific, and HFI-specific rhythmic genes. For NC-specific rhythmic transcripts, the phase of the 1529 transcripts was mainly distributed from ZT0 to ZT7 in the light cycle and from ZT15:30 to ZT21:30 in the dark cycle (µ = 21:29; r = 0.304) (Fig. 5E). The phase of the 1974 HFI-specific rhythmic transcripts was mainly distributed from ZT1:30 to ZT8:30 in the light cycle and from ZT15:30 to ZT20 in the dark cycle (µ = 03:01; r = 0.169) (Fig. 5F). The phase of the 551 shared rhythmic genes was distributed from ZT0 to ZT8 in the light cycle and from ZT12 to ZT24 in the dark cycle (µ = 00.10; r = 0.249) in NC mice (Fig. 5G), and from ZT0 to ZT8 in the light cycle and from ZT15 to ZT20 in the dark cycle (µ = 0.439; r = 0.332) (Fig. 5H) in HFI mice. Notably, 81.3% (448) of the 551 shared circadian transcripts exhibited a phase change by at least 1 hour (Fig. 5I *left*); of these transcripts, 35.0% (193) were phase advanced and 46.3% (255 transcripts) were phase delayed (Fig. 5I *right*). Collectively, these results indicated that rhythmic transcripts tended to be expressed at later phases in the circadian cycle in HFI mice.

On average, the expression amplitude of the 1974 exclusively rhythmic genes in HFI mice (Fig. 5J *right*) was lower than that of the 1529 exclusively rhythmic genes in NC mice (AMP average, 20.17 vs. 8.93; Fig. 5J *left*) (*P* < 0.001). The expression amplitude of the 551 shared rhythmic genes was lower in HFI mice (Fig. 5K *right*) than in NC mice (AMP average, 15.75 vs. 11.47; Fig. 5K *left*) (*P* < 0.01). Collectively, these data indicated that HFI altered both the phase and amplitude of rhythmic gene expression.

### HFI Reprograms KEGG and PSEA in the Cornea

**Figure 6.**
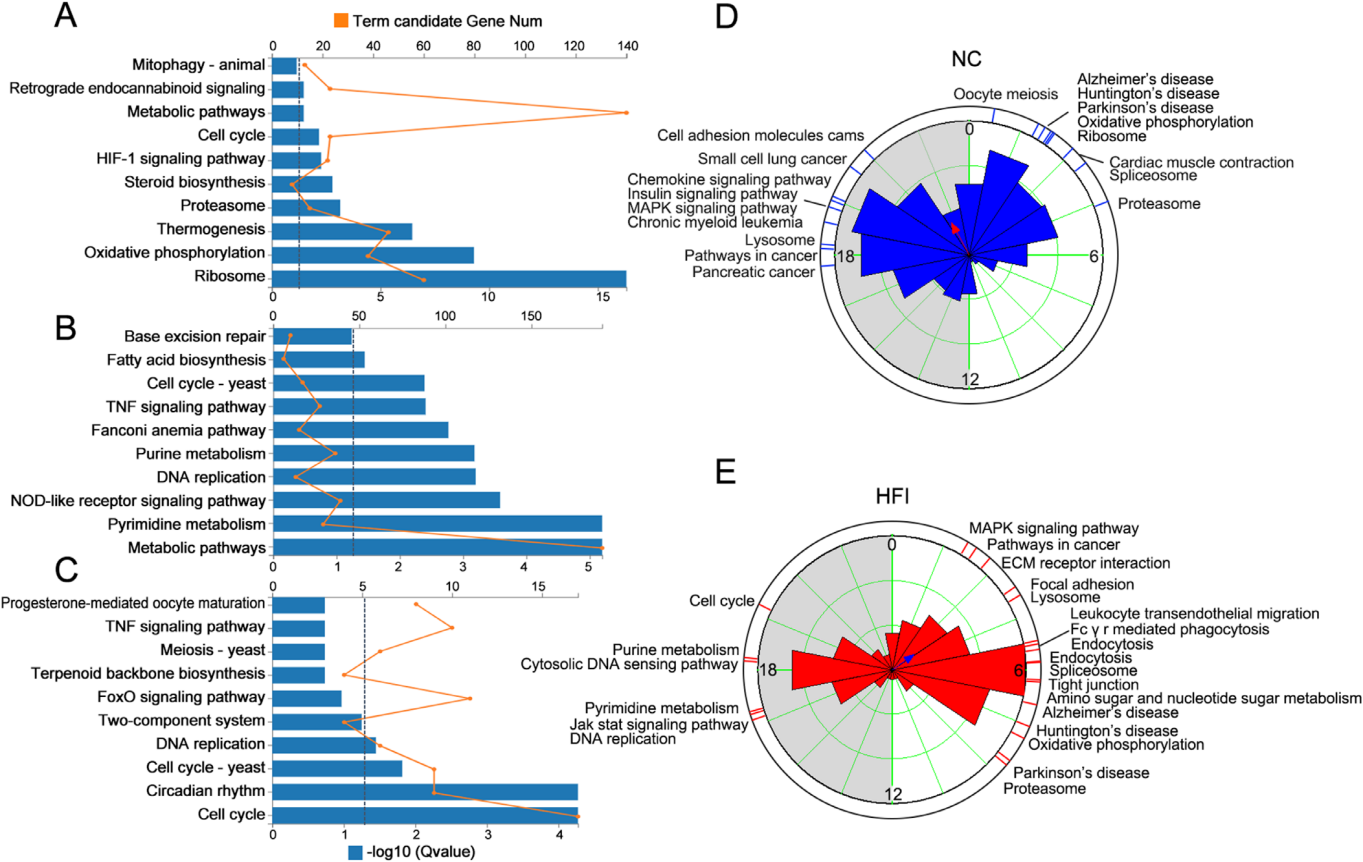
Effects of HFI on phase-clustered pathways in the murine cornea. (**A**) Gene annotation of KEGG pathways enriched for NC-specific oscillating genes with *Q* < 0.05. The top 10 pathways are presented. (**B**) Gene annotation of KEGG pathways enriched for HFI-specific oscillating genes with *Q* < 0.05. The top 10 pathways are presented. (**C**) Gene annotation of KEGG pathways enriched for shared oscillating genes with *Q* < 0.05 in JTK. The top 10 pathways are presented. The horizontal dashed lines in **A**, **B**, and **C** represent the boundary for *Q* < 0.05. (**D**, **E**) Summary of significant phase-clustered (*Q* < 0.01) pathways in the corneas of NC mice (**D**) and HFI mice (**E**). The inner circle depicts the phase distribution of rhythmic genes. The column length represents the number of genes in the phase. The *red dotted line* on the outside circle represents the KEGG pathways (*Q* < 0.01 and >10 genes per pathway) that were enriched at different ZT times based on the phase distribution of the inner circle. The *gray shading* indicates dark cycles.

KEGG enrichment analysis revealed that NC-specific genes were enriched for several metabolic pathways (*Q* < 0.05), including ribosome, oxidative phosphorylation, thermogenesis, proteasome, steroid biosynthesis, heat-inducible factor-1 signaling pathway, and metabolic pathways (Fig. 6A). In contrast, HFI-specific genes were enriched for pathways associated with metabolic pathways, pyrimidine metabolism, NOD-like receptor signaling pathway, DNA replication, purine metabolism, the Fanconi anemia pathway, the TNF signaling pathway, and fatty acid biosynthesis (*Q* < 0.001) (Fig. 6B). Four shared temporally coordinated pathways were identified: cell cycle, circadian rhythm, cell cycle-yeast, and DNA replication (Fig. 6C). These data suggest that HFI reprogrammed rhythmically enriched biological pathways.

We performed PSEA, a novel analytical tool for the visualization of identified and biologically related gene sets with temporally coordinated expression^51^ to examine functional pathways in the corneas of NC and HFI mice that peaked at specific timepoints. The largest number of significant phase clusters (Kuiper *Q* value < 0.01) in the corneas of both groups was observed in the light and dark cycles (ZT0 to ZT6 and ZT18-ZT24 in NC mice; ZT0 to ZT9 and ZT15-ZT21 in HFI mice) (Figs. 6D and E). In total, 18 significantly enriched functional pathways were identified in the corneas of NC mice, grouped into four categories as follows: (1) genetic information processing: proteasome, ribosome; (2) cellular processes: cell adhesion molecules cams, lysosome, oocyte meiosis; ribosome, MAPK signaling pathway, spliceosome; (3) disease-associated pathways: pathways in cancer, Parkinson's disease, Alzheimer's disease, Huntington's disease, pancreatic cancer, small cell lung cancer, chronic myeloid leukemia, oocyte meiosis; and (4) organismal systems: chemokine signaling pathway, insulin signaling pathway, cardiac muscle contraction (Fig. 6D, Supplementary Table S3). In total, 23 significantly enriched functional pathways were identified in the corneas of HFI mice, grouped as follows: (1) metabolic pathways: oxidative phosphorylation, amino sugar and nucleotide sugar metabolism, pyrimidine metabolism, purine metabolism, and glycerophospholipid metabolism; (2) cellular processes, including lysosome, focal adhesion, tight junction, endocytosis, and cell cycle; (3) genetic information processing: spliceosome, DNA replication, and proteasome; (4) organismal systems: extracellular matrix receptor interaction, JAK–STAT signaling pathway, leukocyte transendothelial migration, and FcγR-mediated phagocytosis; and (5) disease-associated pathways, including pathways in cancer (NC group vs. fructose group, ZT18.21/ZT2.26), Parkinson's disease (v ZT2.27/ZT8.47), Alzheimer's disease (ZT1.89/ZT6.85), and Huntington's disease (ZT2.07/ZT7.44) (Fig. 6E, Supplementary Table S4). Each disease-associated pathway was activated in NC and HFI mice but at different ZTs. Collectively, these results indicated that HFI temporally altered the quality and distribution of phase set–enriched signaling pathways.

### HFI Reprograms Cluster-Dependent Transcriptomic Maps in the Cornea

**Figure 7.**
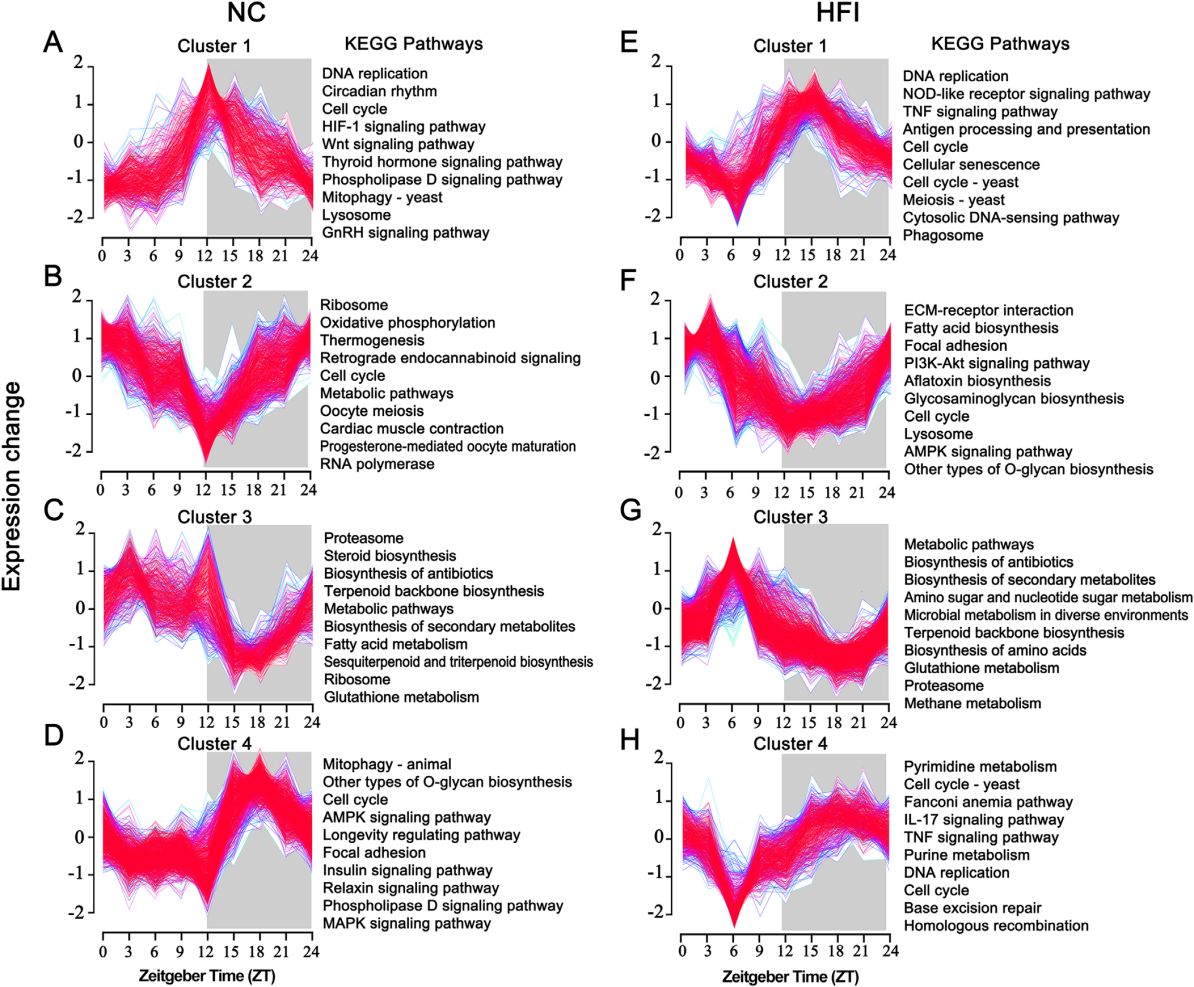
Effects of HFI on circadian transcriptional clustering profiles in the murine cornea. (**A**–**D**) Temporal gene Z-scores from four different enriched clusters in the corneas of NC mice (*left*). Selected enriched KEGG annotations for each cluster are presented (*P* < 0.05) (*right*). (**E–H**) Temporal gene Z-scores from four different enriched clusters in the corneas of HFI mice (*left*). Selected enriched KEGG annotations for each cluster are presented (*P* < 0.05) (*right*). Expression values were standardized to have a mean of 0 and 1 standard deviation. The *blue and green lines* represent low-membership value genes. The red lines represent high-membership value genes. The **gray shading** indicates dark cycles.

Clustering analysis is a powerful visualization tool for biological pathways underlying large gene expression datasets.^52^^,^^53^ To determine the dynamic patterns of transcriptomic activity over a circadian cycle, we performed soft Mfuzz clustering on standardized log2-normalized FPKM values for each sample. Based on the distribution of temporal oscillation peaks and troughs over a 24-hour cycle, we defined four different oscillation clusters (Figs. 7A–H). For cluster 1 (NC vs. HFI groups; 387/484 enriched genes), the peak was located in the light cycle and the trough in the dark cycle. For cluster 2 (580/898 enriched genes), the trough was located at the junction of the light and dark cycles. For cluster 3 (356/481 enriched genes), the peak was located at the junction of the light and dark cycles. For cluster 4 (757/662 enriched genes), the peak was located in the light cycle.

To further visualize the unique functional and biological relevance of each transcriptional cluster, KEGG-enriched analysis was performed (Supplementary Tables S5 and S6). In NC mice, cluster 1 was related to biosynthesis and metabolic pathways (Figs. 7A and B); cluster 2 was related to cellular processes and genetic information processing, including the cell cycle and oocyte meiosis; cluster 3 pathways predominantly comprised signaling pathways, with the exception of the circadian rhythm pathway (Fig. 7C); and cluster 4 pathways were also predominantly associated with signaling pathways (Fig. 7D). Despite similar cluster modes in NC and HFI mice, a KEGG analysis of HFI mice revealed distinct biological pathway annotations (Figs. 7E–H). In clusters 3 and 4, inflammation-associated pathways including Th17, TNF, and NOD-like receptor signaling pathways were identified, suggesting that short-term HFI activated inflammatory events in the cornea. Collectively, these data indicated that HFI reprogrammed dynamic transcriptional clustering of these biological pathways.

### The Core-Clock Gene Transcription in the Cornea Is Not Altered by HFI

**Figure 8.**
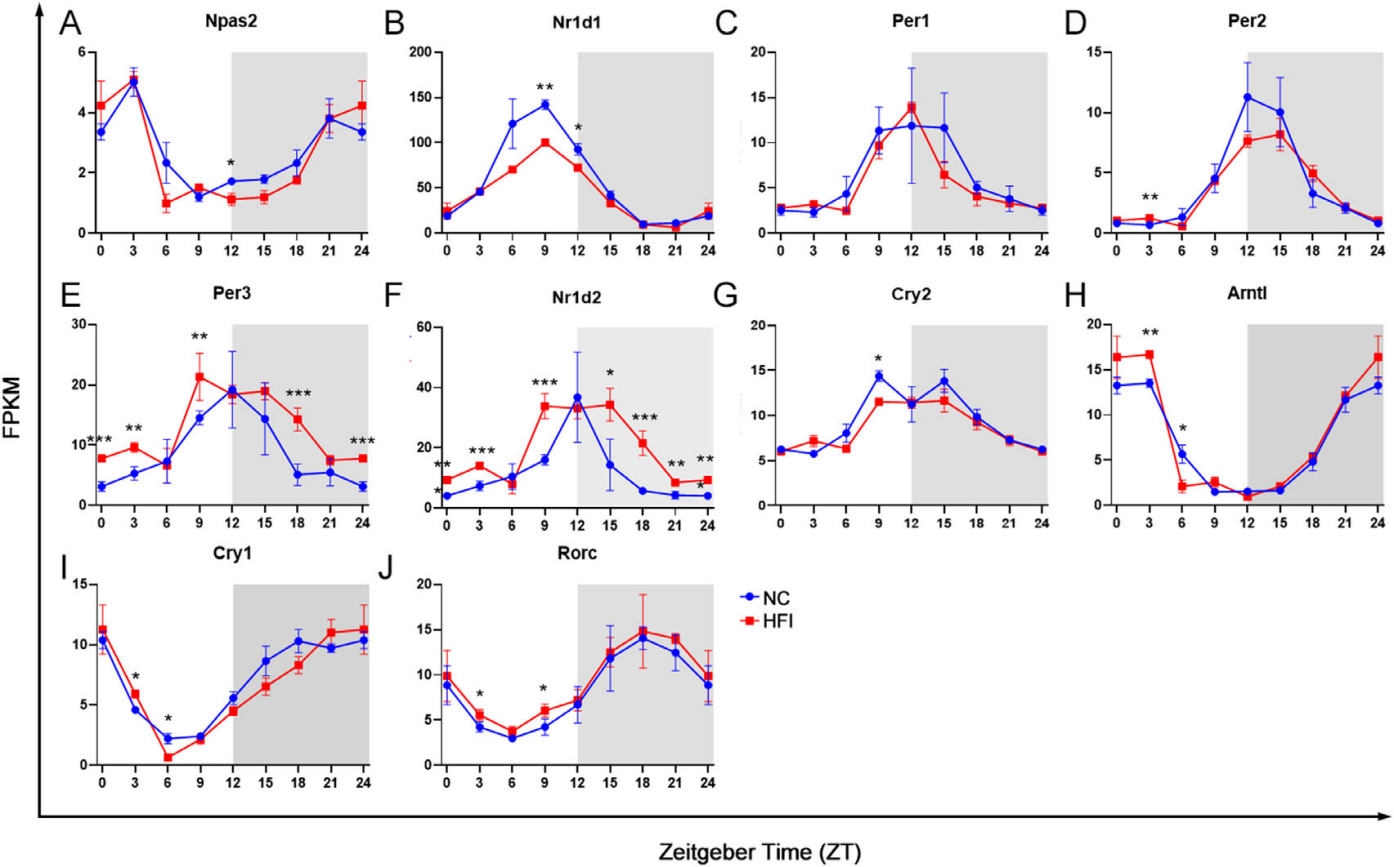
Effect of HFI on circadian transcription of canonical clock genes in the murine cornea. Temporal expression profiles of canonical clock genes were measured in cornea samples collected from three mice at each time point. (**A–J**) The temporal abundance of the transcripts of 10 core-clock genes. The *x*-axis represents the sampled time points. The *y*-axis represents the expression of the genes at specific ZT time points. The gray shading indicates dark cycles. *P < 0.05, **P < 0.01, ***P < 0.001 between groups at each time point.

Changes in the light cycle, such as in the jet lag model, alter the expression of core-clock machinery genes in the normal murine cornea.^11^^,^^33^^,^^35^ To identify the effects of HFI on core-clock machinery genes in the cornea, we analyzed the temporal transcription profiles of ten canonical core-clock genes in the corneas of NC and HFI mice. In NC mice, all canonical core-clock genes exhibited a robust rhythm, two of which (*Npas2* and *Nr1d1*) peaked during the light cycle (ZT3 and ZT9, respectively) (Figs. 8A and B), 5 of which (*Per1, Per2, Per3, Nr1d2,* and *Cry2*) peaked at ZT12 (the transition point of the light to dark cycle) (Figs. 8C–G), 2 of which (*Arntl and Cry1*) peaked at ZT0 (24) (the transition point of the dark to light cycle) (Figs. 8H and I), and only one of which (*Rorc*) peaked during the dark cycle (ZT18) (Fig. 8J). In HFI mice, all 10 canonical core-clock genes also exhibited a robust rhythm with the exception of small but significant changes in the amplitude and peak of *Nr1d1, Per3,* and *Nr1d2* transcription (Figs. 8B, E, and F). Collectively, these data suggested that HFI did not significantly modulate oscillations in core-clock machinery in the cornea.

### HFI Alters Rhythmic Mitosis in the Corneal Epithelium and Cell Growth-Associated Transcriptional Profile

**Figure 9.**
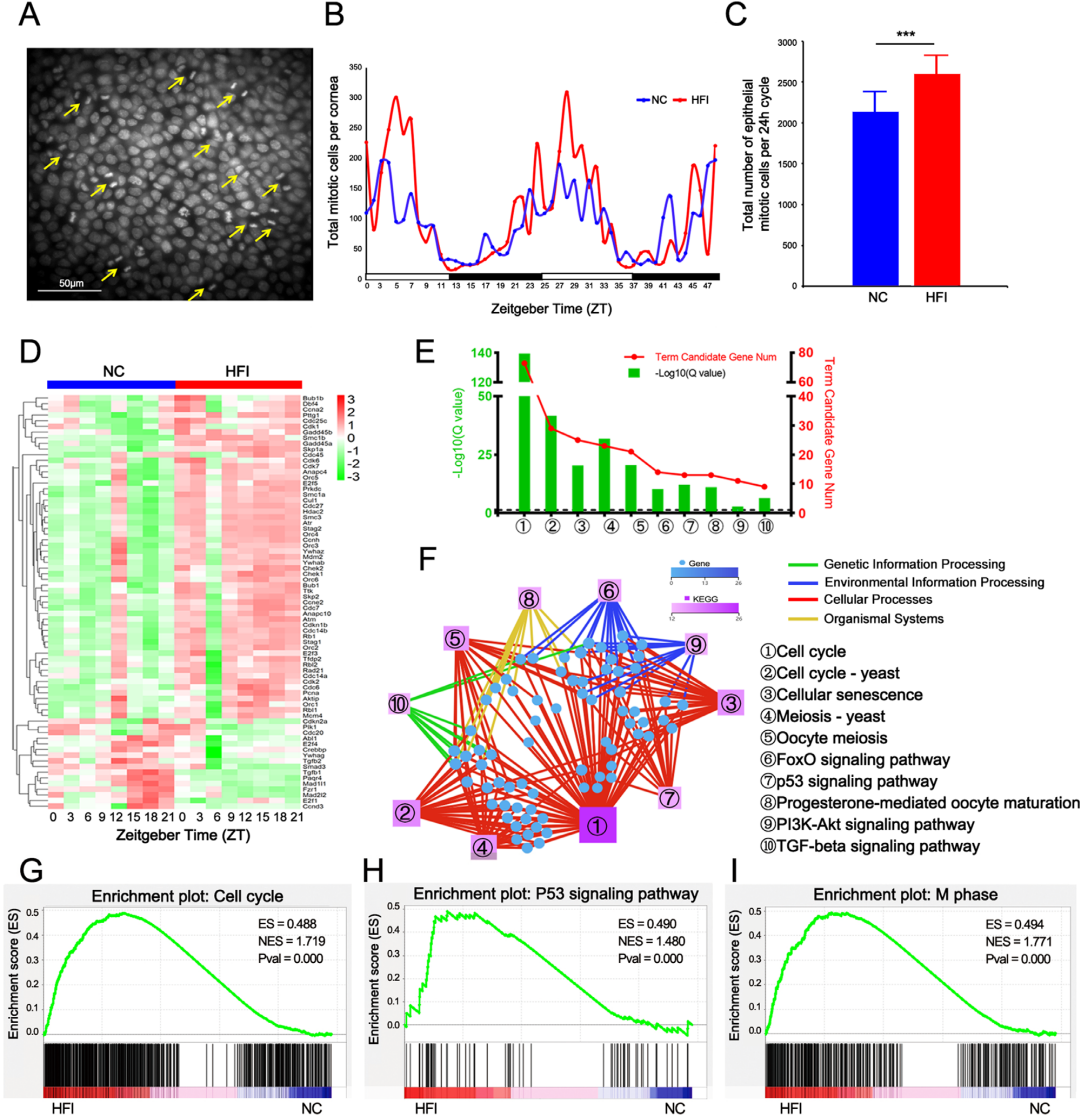
Effects of HFI on mitotic division in the corneal epithelium and expression of cell cycle-associated transcription and signaling pathways over a circadian cycle. (**A**) Representative photomicrograph of mitotic cells in the epithelium with paired nuclei (DAPI staining, *yellow arrows* indicated). Scale bar, 50 µm. (**B**) Distribution of the number of mitotic corneal epithelial cells over two circadian cycles for 48 hours. The average of the sum of mitotic cells quantified in nine vertical and horizontal 40× fields at each time point with six corneas collected at 3-hour intervals. The *blue and red lines* indicate NC and HFI groups, respectively. (**C**) The total number of mitotic cells quantified per 24-hour cycle. ***P < 0.005, *n* = 8 corneas per group. (**D**) RNA-seq analysis of corneal transcripts on day 10 in NC and HFI mice across a circadian cycle. The heatmap displays the differentially expressed transcriptional profiles of the 48 genes related to the cell cycle in HFI and NC mice. (**E**) Gene annotation of KEGG pathways enriched for the differentially expressed transcriptional profiles in the corneas of HFI mice with *Q* < 0.05. The top 10 pathways are presented. (**F**) Cell cycle-associated KEGG network diagram for the HFI group. The *circles* represent transcripts. Color and size indicate the number of genes or transcripts connected to each node. The darker the color and the larger the box, the more genes or transcripts connected to the node. Different colored lines represent different pathway classifications. The *red, blue, green, and yellow lines* represent cellular processes environmental information processing, genetic information processing, and organismal systems, respectively. (**G–I**) GSEA results of enrichment plots for cell cycle-related pathways in the corneas of HFI mice, including gene sets related to the cell cycle, p53 signaling pathway, and M phase. The *left red* part and the *right blue* part of the heatmap at the bottom represent the high expression of the corresponding functional pathway genes in the HFI group and NC group, respectively.

The cornea is highly regenerative^56^ and mitotic division in the corneal epithelium exhibits diurnal rhythms.^33^ To examine the effects of HFI on diurnal mitotic patterns in the corneal epithelium, we quantified limbus-to-limbus mitotic cell number. We observed that the active phase for epithelial division occurred from ZT17 to ZT33 and peaked around ZT6 in NC mice, in agreement with our previous reports.^33^ The amplitude and total number of mitotic cells over a circadian cycle were significantly higher in HFI mice than in NC mice (Figs. 9A–C).

We next analyzed transcriptional alterations in cell cycle, growth, and differentiation-associated transcripts over a circadian cycle. In total, 73 differentially expressed transcripts were identified (>I±I 0.3 fold) (Fig. 9D). Enrichment analysis of these differentially expressed transcripts revealed that the top 10 KEGG pathways were associated with the cell cycle, cellular senescence, meiosis, and FoxO-, p53-, and TGF-β–related signaling pathways (Fig. 9E). The relationships between selected rhythmic genes and KEGG pathways are depicted in Figure 9F and Supplementary Table S7. Based on the ES of GSEA of all transcripts, the REACTOME cell cycle, M phase, and KEGG p53 signaling pathways were enriched in HFI mice (Figs. 9G–I). Collectively, these data suggested that HFI induced dysfunction in mitotic rhythms and associated transcriptional profiles in the corneal epithelium.

### HFI Alters Immune Cell Trafficking to the Corneal Limbus and Immune-Associated Transcription

**Figure 10.**
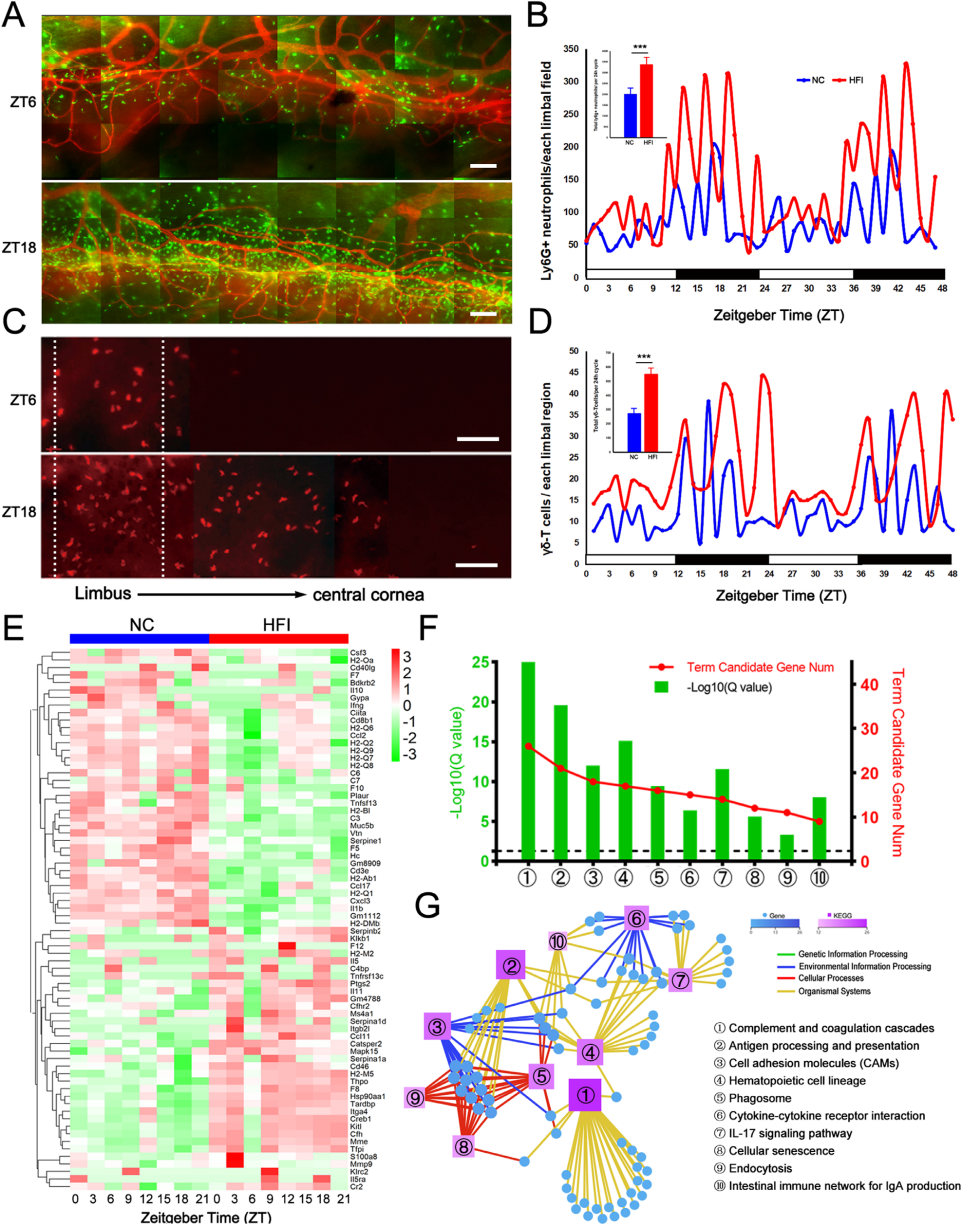
Effect of HFI on circadian trafficking of neutrophils and γδ-T cells to the limbal region and immune-associated transcriptomic profiles in the murine cornea. (**A**) Representative images of neutrophils around the limbal vascular network at ZT6 and ZT18 in corneas from NC mice; neutrophils (FITC-conjugated anti-Ly6g, *green*), limbal blood vessels (PE-conjugated anti-mouse CD31, *red*), scale bar = 100 µm. (**B**) Changes in the number of neutrophils in the limbus over two circadian cycles from eight corneas collected every hour for 48 hours (ZT scale). The *blue and red lines* represent NC and HFI groups, respectively. The *top* *left inset* depicts the total number of neutrophils quantified per 24-hour cycle. ***P < 0.005, n = 8 corneas per group. (**C**) Representative images of γδ-T cells around the limbal and paralimbal area at ZT6 and ZT18 in corneas of NC mice; γδ-T cells (PE-conjugated anti–TCRδ; *red*); scale bar = 40 µm. (**D**) Changes in the number of γδ-T cells in the limbal and paralimbal area over two circadian cycles from eight corneas collected every hour for 48 hours (ZT scale). The *blue and red lines* represent the NC and HFI groups, respectively. The *top* *left inset* depicts the total number of γδ-T cells quantified per 24-hour cycle. ***P < 0.005, *n* = 8 corneas per group. (**E**) RNA-seq analysis of corneal transcripts on day 10 in NC and HFI mice over a circadian cycle. The heatmap displays expression levels of the 48 genes related to immunity that were significantly differentially expressed in HFI mice compared with NC mice. (**F**) Gene annotation of KEGG pathways enriched in the corneas of HFI mice on day 10 with *Q* < 0.05. The top 10 pathways are presented. The horizontal dashed line in the figure represents the boundary for *Q* < 0.05. (**G**) Immune-associated KEGG network diagram for the HFI group. The *boxes* and *circles* represent KEGG pathways and transcripts, respectively. Color and size indicate the number of genes or transcripts connected to each node. The darker the color and the larger the box, the more genes or transcripts connected to a node. The different colored lines represent different pathway classifications. The *blue, yellow, and red lines* represent environmental information processing, organismal systems, and cellular processes, respectively.

Neutrophils and γδ-T cells are the essential innate immune cells on the ocular surface of mice and play an important role in maintaining the healthy state of ocular surface and pathological reaction.^57^^–^^59^ However, it is not clear whether HFI alters the diurnal trafficking of neutrophils and γδ-T cells to the cornea. In the corneas of NC mice collected at hourly intervals, the active trafficking of neutrophils and γδ-T cells to the corneal limbal region occurred during the dark cycle and peaked at ZT18 (Figs. 10A–D). Trafficking of neutrophils and γδ-T cells to the cornea and total cell number over a circadian cycle were significantly greater in HFI mice than in NC mice (Figs. 10A–D).

Differentially expressed transcripts in the corneas of HFI and NC mice over a circadian cycle (>±1 fold) are presented in the heatmap in Figure 10E. KEGG enrichment analyses revealed that the top 10 enriched pathways were related to immune-associated functions (Fig. 10F). The relationships between these KEGG pathways and selected rhythmic genes are presented in Figure 10G. A complete list of the most overrepresented KEGG pathways in both groups is presented in Supplementary Table S8. Our data suggested that HFI promoted immune cell recruitment to the limbal region and induced alterations in immunologic pathways in the murine cornea over a 24-hour circadian cycle.

### HFI Alters Metabolic Pathways in the Cornea

**Figure 11.**
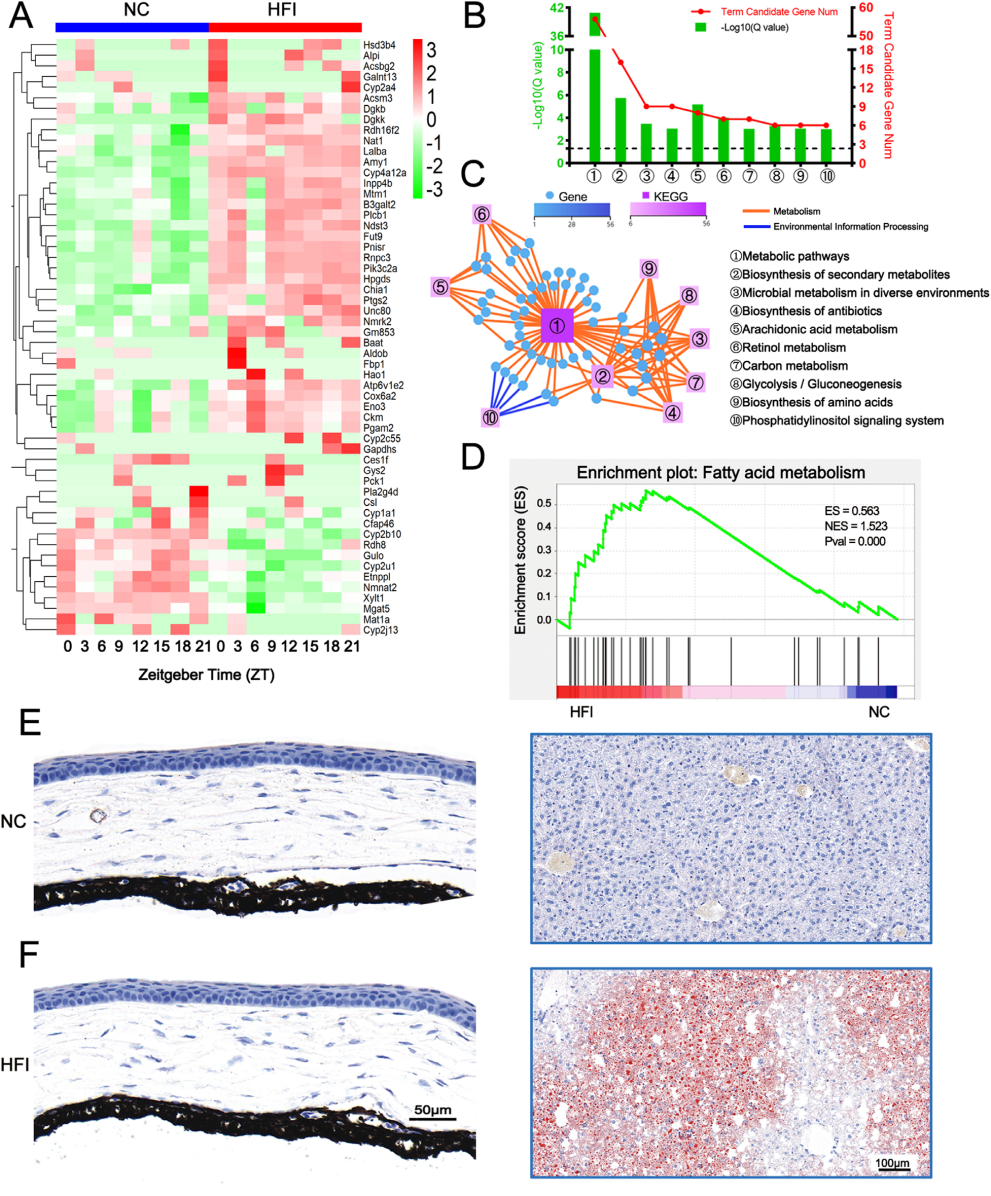
Effects of HFI on metabolism-associated transcriptomic profile and signaling pathways. (**A**) RNA-seq analysis of corneal transcripts on day 10 in NC and HFI mice over a circadian cycle. The heatmap displays expression levels of the 56 genes related to metabolism that were significantly differentially expressed in HFI mice compared with NC mice. (**B**) Gene annotation of KEGG pathways enriched in the corneas on day 10 after HFI with *Q* < 0.05. The top 10 pathways are presented. The horizontal dashed line in the figure represents the boundary for *Q* < 0.05. (**C**) Metabolism-associated KEGG network diagram in the corneas of HFI mice. The *boxes* and *circles* represent KEGG pathways and transcripts, respectively. Color and size indicate the number of genes or transcripts connected to each node. The darker the color and the larger the box, the more transcripts connected to a node. The different colored lines represent the different pathway classifications. The *blue and orange lines* represent environmental information processing and organismal systems, respectively. (**D**) GSEA results of enrichment plots for the KEGG_Fatty_Acid Metabolism pathway in the corneas of HFI mice. The *left red* part and the *right blue* part of the heatmap at the bottom represent the high expression of the corresponding functional pathway genes in the HFI group and NC group, respectively. (**E**, **F**) Representative images of Oil Red O histological stains of cross-sections of the cornea and liver from an NC mouse (**E**) and HFI mouse (**F**) on day 10. Scale bars, 50 µm (cornea) and 100 µm (liver).

Liver metabolites of HFI are converted into fat and enter the blood circulation, inducing metabolic stress to the body.^3^ Corneal tissues are distant to blood vessels and possess unique metabolic pathways.^60^ To examine the effects of HFI on corneal metabolism under normal physiological conditions, we analyzed the corneal metabolism-related transcriptome over a 24-hour cycle after 10 days of HFI. HFI significantly altered the expression of many metabolism-related transcripts (Fig. 11A). Interestingly, we found that there were many members of the cytochrome P450 enzyme superfamily in the transcripts of differential expressions, including *CYP2a4*, *4a12a, 2c55, 1a1, 2b10, 2u1,* and *2j13* (Fig. 11A). Enrichment analysis for these differentially expressed transcripts revealed that the main enriched signaling pathways were related to biosynthesis, microbial metabolism, arachidonic acid metabolism, retinol metabolism, carbon metabolism, and glycolysis and gluconeogenesis (Fig. 11B). The relationships between these KEGG pathways and selected rhythmic genes are presented in Figure 11C and Supplementary Table S9. GSEA revealed that the KEGG_fatty acid metabolism pathway was enriched specifically in the corneas of HFI mice (Fig. 11D). In HFI mice, mild lipid deposits were observed in the liver but not in the cornea (Figs. 11E and F). These data collectively indicated that HFI altered metabolic pathways, especially those related to fatty acid metabolism, in the murine cornea.

### HFI Alters Neural Activity in the Cornea

**Figure 12.**
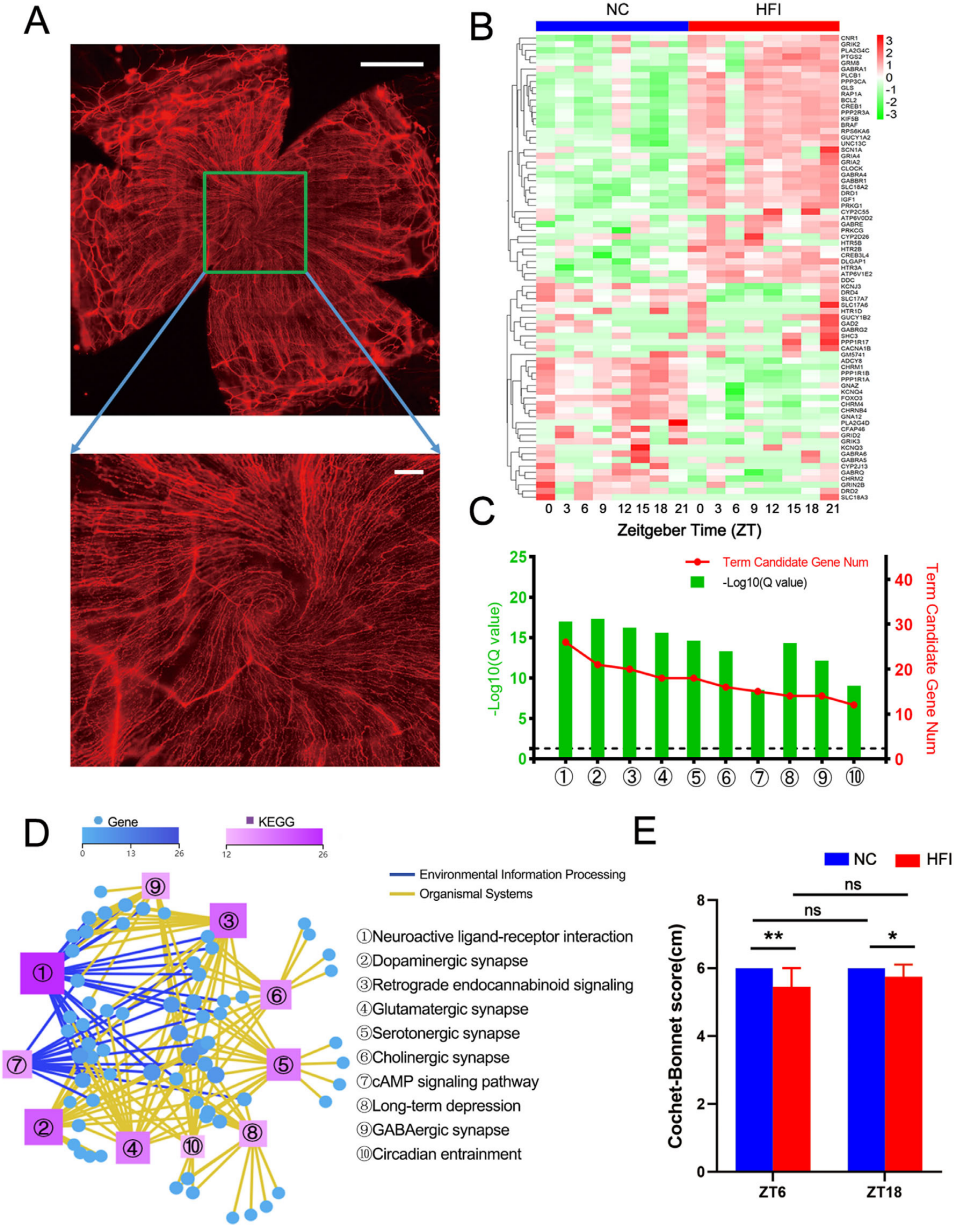
Effects of HFI on corneal neural activity. (**A**) Representative image of a cornea from a mouse sacrificed at ZT12. The cornea was stained with antibodies against β-III tubulin to reveal the sub-basal nerves. The images were stitched with the whole cornea (upper panel) and 6 × 8 individual images (*bottom*) using a DeltaVision Elite microscope under 40× magnification. Image scale bar, 500 µm (*top*) and 60 µm (*bottom*). (**B**) RNA-seq analysis of corneal transcripts on day 10 in NC and HFI mice over a circadian cycle. The heatmap displays expression levels of the 75 transcripts related to neural activity that were significantly differentially expressed in the corneas of HFI mice compared with NC mice. (**C**) Gene annotation of KEGG pathways enriched in the corneas on day 10 after HFI with *Q* < 0.05. The top 10 pathways are presented. The horizontal dashed line in the figure represents the boundary for *Q* < 0.05. (**D**) Neural activity-associated KEGG network diagram for the corneas of HFI mice. The *boxes* and *circles* represent KEGG pathways and transcripts, respectively. Color and size indicate the number of genes or transcripts connected to each node. The darker the color and the larger the box, the more genes or transcripts connected to a node. The different colored lines represent the different pathway classifications. The *blue and yellow lines* represent environmental information processing and organismal systems, respectively. (**E**) Corneal sensitivity was measured using a Cochet–Bonnet esthesiometer and compared between NC and HFI mice. The results are presented as the mean ± standard deviation. *P < 0.05, **P < 0.01, *n* = 10 corneas per group.

HFI causes metabolic stress^3^ and may trigger nervous system abnormalities in combination with other factors such as uric acid (a fructose metabolite).^61^ The cornea is densely innervated by sensory nerve fibers (Fig. 12A) and is highly sensitive to external stimuli, especially mechanical stimuli. To explore the effects of HFI on corneal sensory function, we analyzed nerve-related transcripts at eight time points in a circadian cycle and identified 75 nerve-related transcripts that were differentially expressed in the cornea (>I±I 1 fold) (heatmap in Fig. 12B). An enrichment analysis of these differentially expressed transcripts revealed the top 10 pathways with significant changes (Figs. 12C and D). The remaining pathways are listed in Supplementary Table S10. To verify the functional correlates of these transcriptomic changes, we measured changes in corneal sensitivity using a Cochet–Bonnet esthesiometer. No significant difference in corneal sensitivity was observed in either NC mice or HFI mice at ZT6 and ZT18. However, corneal sensitivity was significantly lower in HFI mice than in NC mice at both ZT6 and ZT18 (Fig. 12E). Collectively, these data indicated that HFI altered corneal neural activity at molecular and functional levels.

## Discussion

**Figure 13.**
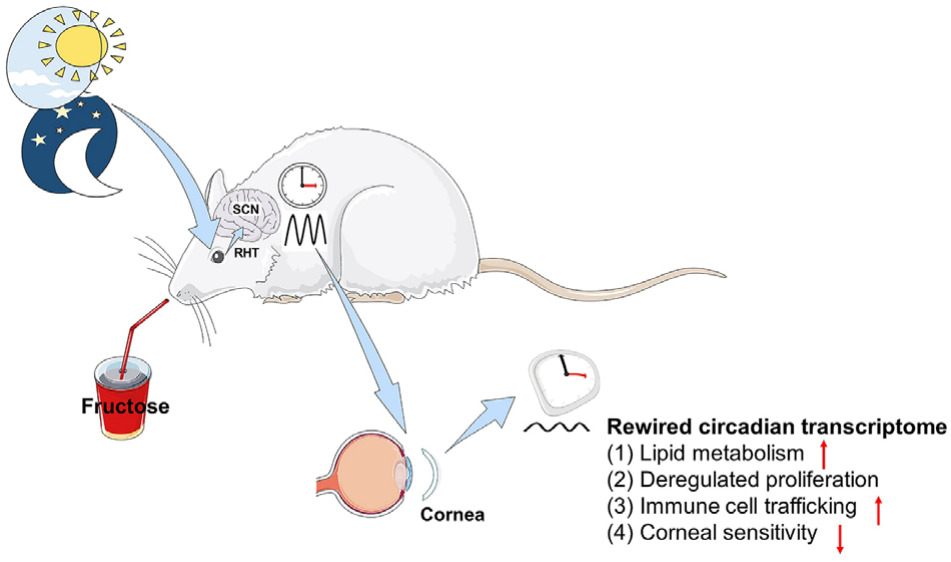
Schematic of rewired diurnal fluctuations in the corneas of HFI mice. The central clock system in the SCN is regulated by light via the RHT. Short-term HFI reprograms the corneal molecular clock, causing dysregulated proliferation, increased immune cell trafficking, increased lipid metabolism, and decreased corneal sensitivity. RHT, retino-hypothalamic tract.

Similar to most mammalian organs, the cornea exhibits robust circadian rhythms in various physiological functions, including diurnal recruitment of white blood cells into the corneal limbus and epithelial cell mitosis.^33^^–^^36^ However, the molecular mechanisms underlying these circadian rhythms are poorly understood. Our latest published data revealed daily fluctuations in transcriptomic profiles in the murine cornea to adapt to light and dark phases.^35^ Here, we provide a comprehensive analysis of the temporal and spatial distributions of the circadian transcriptome in murine cornea under a normal light/dark cycle. Because these animals were under a normal light/dark cycle, the observed transcriptomic changes of core clock genes were unaffected by the traditional light–retina–SCN axis, consistent with transcriptomic changes of core clock genes in the liver caused by Western diets.^29^^,^^30^^,^^62^ Notably, we observed that short-term HFI rewired the circadian transcriptome in the cornea under unaltered light, especially in pathways associated with metabolism, mitosis, neural activity, and immune function. We identified significant alterations in temporally coordinated and cluster-dependent transcriptomic landscapes of circadian transcripts in the cornea after 10 days of HFI. Further, we determined that HFI significantly altered the normal circadian recruitment of neutrophils and γδ-T cells to the cornea, predominantly in the limbal area, as well as the pattern and number of mitotic divisions in the corneal epithelium over a circadian cycle. These results highlight novel pathological alterations in the cornea induced by HFI (Fig. 13).

Circadian rhythms and energy metabolism are closely interlinked.^63^ Environmental and genetic perturbations to circadian rhythms contribute to metabolic dysfunction.^64^^–^^66^ In turn, the circadian system senses metabolic cues. Perturbations to metabolism such as high-calorie diets or altered feeding times disrupt the temporal coordination of circadian rhythms and may lead to various disorders, including obesity, diabetes, and sleep problems.^67^^–^^69^ Here, we used excessive fructose consumption as an exogenous metabolic challenge and observed significant rewiring of oscillations in circadian gene transcription and associated signaling pathways in murine cornea over the light/dark cycle. Further, the newly generated rhythmic genes in the corneas of HFI mice were derived from nonrhythmic genes in normal corneas, resembling the pattern of de novo transcriptional oscillations that we previously observed in murine extraorbital lacrimal glands after short-term HFI.^11^

Bioinformatics analysis of high-throughput RNA-seq data is a powerful approach to elucidate the complex molecular mechanisms underlying circadian processes.^70^ We assessed the metabolic and signaling pathways of circadian genes affected by excessive fructose intake using a combination of KEGG, PSEA,^51^ and time series clustering approaches.^52^^,^^53^ We observed significant alterations in metabolic and other signaling pathways alongside changes in the time-phase distributions of these pathways in the corneas of HFI mice. These circadian transcriptomic changes provide insight into HFI-induced corneal pathogenesis. Nevertheless, the precise mechanisms of dysregulation require further in-depth analysis via proteomics and metabolomics.

Every mammalian cell contains a set of core-clock genes that govern downstream clock-controlled genes via self-regulatory transcriptional and translational feedback loops.^71^^,^^72^ Transcriptional alterations in core-clock genes may drastically affect physiological functions. We observed that the transcription of core-clock genes in the cornea was resistant to excessive fructose consumption, consistent with our previous findings in the extraorbital lacrimal glands in a short-term HFI model^11^ and in the liver after high-fat diet treatment.^29^ Collectively, these data support the concept that circadian oscillations in core-clock genes are highly resistant to metabolic challenges.^29^^,^^67^^,^^68^ Nevertheless, the effects of the nutritional state on the regulation of circadian clocks remain to be defined.

Similar to other epithelial tissues, the corneal epithelium undergoes constant renewal, which occurs over 5 to 7 days.^73^ Turnover is mainly accomplished by limbal epithelial stem cells located at the limbus and the proliferation and migration of limbal epithelial stem cell–differentiated transient amplifying cells.^73^^–^^76^ Recent evidence has indicated that the cell cycle is controlled by circadian rhythms.^77^ Studies from our group and other groups have demonstrated that corneal epithelial mitosis exhibits a significant diurnal oscillation.^33^^–^^36^^,^^78^^–^^81^ Recent studies suggest that circadian rhythms synchronize with the regular light/dark cycle and are retrained by nonphotic zeitgebers such as food and feeding, ambient temperature, social contact, and physical activity.^82^ Consistent with these data, we observed that HFI activated cell cycle-related signaling pathways in the cornea, especially cell cycle checkpoints, M phase, and p53 signaling pathways. In accordance with these transcriptional changes, we observed that the corneal epithelial mitotic cell number was significantly increased over a circadian cycle. Our data support recent reports of the close association between high fructose consumption and a high incidence of cancer.^83^^,^^84^ Collectively, these results suggest that even short-term HFI rapidly activates cell cycle-related molecular mechanisms in the cornea.

With the exception of the limbal area, the cornea is an avascular tissue. To maintain rapid corneal epithelial turnover, high sensitivity, and transparency, the cornea obtains energy via unique metabolic pathways.^85^ For instance, oxygen supply to the cornea arises predominantly from the atmosphere in the open eye and tarsal conjunctival capillaries in the closed eye.^86^ Most nutrients, including glucose, are diffused from the aqueous humor and tear film.^87^ However, the effects of HFI on normal corneal metabolism have not been reported. Our transcriptomic data indicated that various metabolic-related transcripts were differentially expressed in the cornea after HFI; in particular, transcripts of fatty acid metabolism pathways were significantly enriched. Consistent with these results, we identified the high expression of several cytochromes P450 family members in the corneas of HFI mice. Cytochromes P450s constitute an enzyme superfamily that oxidizes fatty acids, xenobiotics, and various compounds for clearance.^88^

The cornea receives the highest innervation density in the human body,^89^ with several sensory fiber subtypes that sense distinct external stimuli.^90^^,^^91^ Neural activity in the cornea is diurnal, including corneal sensitivity in humans,^92^ sensory axon growth and shedding in the murine cornea,^93^ and sensations in individuals wearing contact lenses.^94^ We observed that corneal sensitivity was significantly decreased after HFI. Multiple reasons may underpin this observation. First, as demonstrated by our enrichment analysis of neural activity-related transcripts, many synaptic pathways associated with nerve conduction were altered significantly. Second, a large number of immune cells, including neutrophils and γδ-T cells, were recruited around the limbus blood vessels after HFI. These factors, in combination with metabolic stress and energy alterations, may have decreased conduction speed and neural activity after HFI.^61^^,^^95^

During a 24-hour circadian cycle, immune cells are periodically released from the bone marrow to the peripheral blood^96^ and migrate from the blood to peripheral organs and tissues.^97^^,^^98^ We previously reported that neutrophils and γδ-T cells migrate rhythmically to the corneal limbal region.^36^ The current data revealed a significant increase in the number of recruited immune cells to the corneal limbus after HFI, suggesting that nutritional challenges modulate the plasticity of corneal immune function. These effects may alter the degree and status of the corneal response to various external stimuli such as injuries and microbial infections.

Our study has a few limitations. It should be noted that we only collected murine corneas 10 days after HFI. Long-term HFI or fructose consumption in combination with other nutrients such as lipids may reveal distinct insights into the effects of high-calorie diets on corneal function.^99^ In addition, our bioinformatics analysis was limited to high-throughput RNA-seq analysis, which does not yield information on translational, post-translational, or proteomic regulation. Future in-depth analyses of corneal structure and function alongside other -omics approaches are warranted to dissect pathologic mechanisms in the cornea induced by excessive fructose intake.

## Conclusions

Our findings suggest that short-term excessive fructose intake significantly rewires diurnal oscillations in the cornea with regards to corneal epithelial mitosis, immune cell recruitment to the corneal limbus, and transcriptomic profiles. Our findings imply that metabolic challenges induced by HFI alter normal physiological processes in the cornea, including excessive cell proliferation, decreased corneal sensitivity, and a subinflammatory condition. These alterations might modulate the corneal ability to respond to various stimuli from external environments, such as injuries, microbial infection, and desiccation stress. Further analysis of reprogramming mechanisms will reveal potential targets to prevent the onset and progression of pathologic alterations of the cornea induced by excessive fructose consumption. Notably, these data highlight the critical role of nutritional interventions in corneal health.

## Supplementary Material

Supplement 1

Supplement 2

Supplement 3

Supplement 4

Supplement 5

Supplement 6

Supplement 7

Supplement 8

Supplement 9

Supplement 10
